# Deep phenotyping of patient lived experience in functional bowel disorders using machine learning

**DOI:** 10.1038/s41598-025-19273-3

**Published:** 2025-10-09

**Authors:** James K. Ruffle, Michelle Henderson, Cho Ee Ng, Trevor Liddle, Amy P. K. Nelson, Parashkev Nachev, Charles H. Knowles, Yan Yiannakou

**Affiliations:** 1 Queen Square Institute of Neurology, University College London, London, UK; 2 Qufora A/S, Gydevang 30, Allerød, DK-3450 Denmark; 3 Durham Bowel Dysfunction Service, University Hospital North Durham, County Durham and Darlington NHS Trust, Durham, Durham, UK; 4 Research Informatics, Newcastle Hospitals NHS Trust, Newcastle, UK; 5 London School of Medicine and Dentistry, Blizard Institute, Queen Mary University of London, Barts, London, UK

**Keywords:** Deep phenotyping, Functional bowel disorders, Personalised care, Quality of life, Treatment, Artificial intelligence, Machine learning, Graph theory., Gastrointestinal system, Gastrointestinal diseases, Computer science, Statistics

## Abstract

Contemporary clinical management relies on a diagnostic label as the primary guide to treatment. However, individual patients’ lived experiences vary more widely than standard diagnostic categories reflect. This is especially true for functional bowel disorders (FBDs), a heterogeneous and challenging group of syndromes where no definitive diagnostic tests, clinical biomarkers, or universally effective treatments exist. Characterising the link between disease and lived experience - in the face of marked patient heterogeneity - requires deep phenotyping of the interactions between multiple characteristics, plausibly achievable only with complex modelling approaches. In a large patient cohort (*n* = 1175), we developed a machine learning and Bayesian generative graph framework to better understand the lived experience of FBDs. Iterating through 59 factors available from routine clinical care, spanning patient demography, diagnosis, symptomatology, life impact, mental health indices, healthcare access requirements, COVID-19 impact, and treatment effectiveness, machine models were used to quantify the predictive fidelity of one feature from the remainder. Bayesian stochastic block models were used to delineate the network community structure underpinning the heterogeneous lived experience of FBDs. Machine models quantified patient personal health rating (R^2^ 0.35), anxiety and depression severity (R^2^ 0.54), employment status (balanced accuracy 96%), frequency of healthcare attendance (R^2^ 0.71), and patient-reported treatment effectiveness variably (R^2^ range 0.08–0.41). Contrary to the view of many healthcare professionals, the greatest model predictors of patient-reported health and quality of life were life impact, mental well-being, employment status, and age, rather than diagnostic group or symptom severity. Patients responsive to one treatment were more likely to respond to another, leaving many others refractory to all. Clinical assessment of patients with FBDs should be less concerned with diagnostic classification than with the wider life impact of illness, including mental health and employment. The stratification of treatment response (and resistance) has implications for clinical practice and trial design, necessitating further research.

**Supplementary Information:**

The online version contains supplementary material available at 10.1038/s41598-025-19273-3.

## Introduction

Nosology is the branch of medical science that assigns a disease label to a given pathological state. It is fundamentally a classification task in which clinical history, examination, and investigational (such as serology or imaging) features are used to reach a diagnosis. However, there is much more to the individual patient’s experience than simply their disease label. Yet, in contemporary medical research and clinical practice, an objective characterisation of this aspect is rare.

A crucial factor in every disease and every individual patient is the patient’s lived experience^1^which is the knowledge and understanding gained from personally living through something^2^. Clinically, lived experience is typically conceived as merely downstream of critical diagnostic features. However, in many disorders, the patient’s lived experience is tightly interwoven with their diagnosis. Understanding a patient’s lived experience, therefore, merits a close investigation across all available experiential, physiological, and pathophysiological levels.

Functional bowel disorders (FBDs) are a key example here^3,4^. Lived experience (often incorporating many years ahead of a diagnosis being reached^5^ is deeply embedded in symptomatology. These are an exceptionally complex group of diseases with multiple biopsychosocial aspects, rendering them unique to each affected individual. They differ radically from most other gastrointestinal (GI) disorders, where clinical assessment and/or an investigational test can be used to assign a disease label and follow a treatment algorithm. This is arguably a key reason why diagnostic and therapeutic innovation for FBDs has been considerably slower in progress.

Managing patients with FBDs is challenging for many reasons, including marked heterogeneity across patients (and even within the same patient at different times), the absence of precise diagnostic tests and clinical biomarkers, and a diagnostic classification based on symptom profiles that may overlap and change^6^. An incomplete understanding of FBD pathophysiology has held back the development of targeted treatments^7^including the prediction of which patients stand to benefit most from them, leaving this affected group of patients inequitably disadvantaged compared with many other disease groups.

The current diagnostic classification of FBDs is based on varying combinations of GI symptoms^4^. A patient’s experience of FBDs, however, is governed not merely by perturbation of the gastrointestinal tract but has a far-reaching impact across the broader aspects of their life, ranging from the effect on daily activities, mental well-being, access to and satisfaction with healthcare, and treatment efficacy^3,4,6,8–11^. Patients affected by FBDs typically remain so for many years; living with these disorders becomes a necessity, leading to a considerable impact on quality of life (QoL), often with deleterious effects on other aspects of health^8,12^.

While FBDs are multifaceted, high-dimensional disorders interwoven with the patient’s lived experience^13,14^they – like most other diseases - are rarely statistically modelled as such^6^. Clinical research studies often investigate these syndromes within relatively low-dimensional and/or linear statistical frameworks. Common experimental designs, for example, may explore sex differences between disorder *x* or age-related effects of treatment *y*, but rarely provide a comprehensive investigation of multiple factors and their interactions. Such approaches invariably neglect many factors that define the individual, leaving gaps in our understanding of both the patient’s disorder and their lived experience^1^. The same applies to contemporary quantitative measures of lived experience, such as quality of life (QOL) questionnaires, where a complex and multivariate pattern is often condensed into a univariate score^15^. Whilst such an approach is highly accessible, explaining its broad appeal across healthcare, complex models offer substantially greater insight into the whole patient experience, with correspondingly greater actionable and translational potential.

The task requires deep phenotyping of the patient’s lived experience, which has yet to be attempted at the scale and expressivity that FBDs demand. We therefore developed a comprehensive pipeline harnessing machine learning and Bayesian generative graph models to characterise patient lived experience, prototyping it across a large UK cohort of patients with FBDs. By placing the individual patient’s perspective at the forefront of our approach^1^we reveal the associations of ill health, such as the impact on QoL and treatment effectiveness, in a more meaningful and patient-oriented way. This framework could pave the way to more richly individualised patient care^1,16–18^.

## Results

### Cohort

**Fig. 1:**
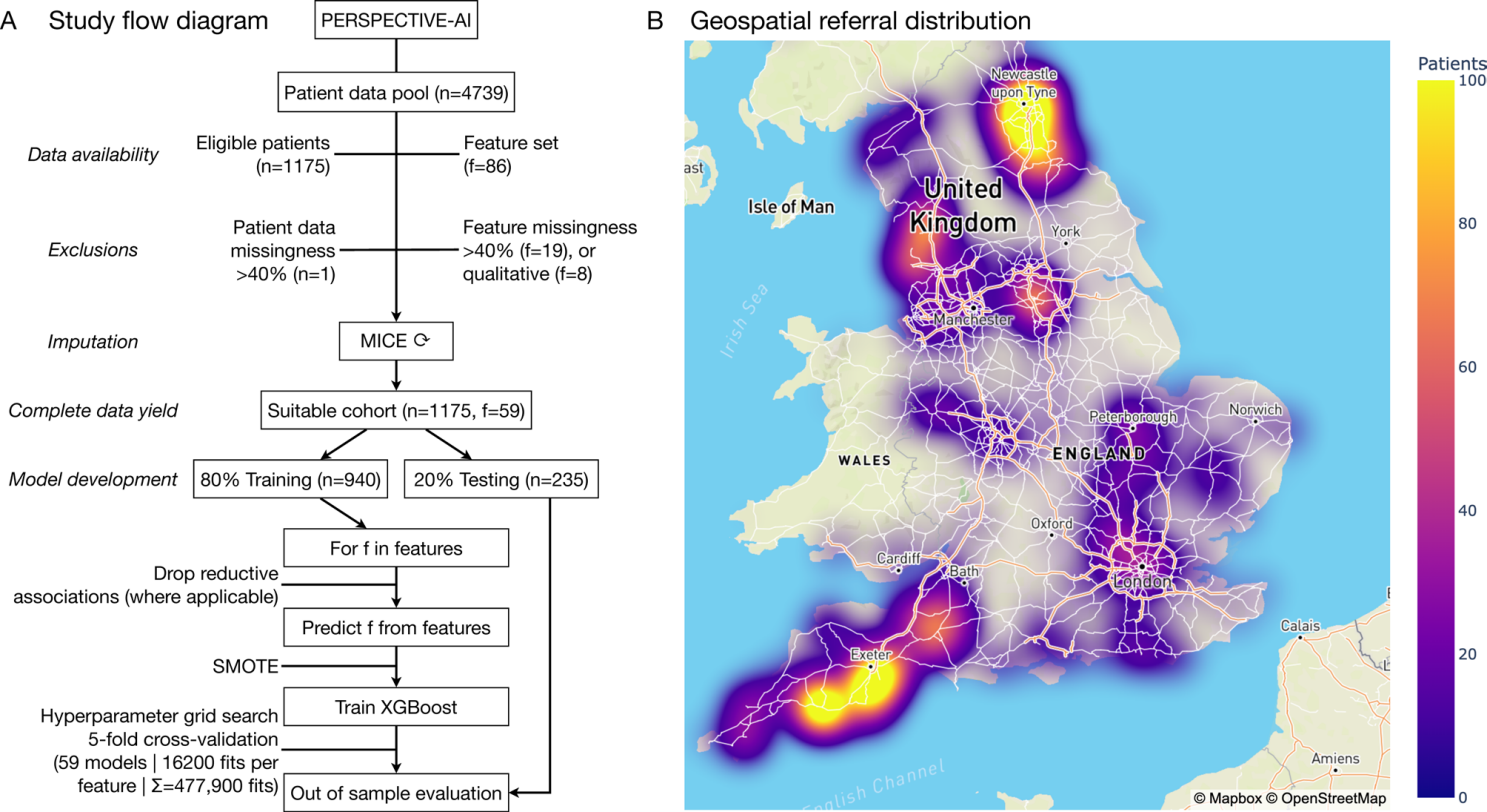
Study design. **A**) Flow diagram. **B)** Geospatial referral distribution. Map generated using OpenStreetMap and Mapbox, the data of which is available under the Open Database License.

We received 1175 responses from 4739 patients (response rate 24.8%), who formed our analysis cohort. The mean age was 52 years (range 20–80 years): female (*n* = 1000) and male (*n* = 175) (Fig. 1). This sex distribution aligns with existing research^19^. 642 patients fulfilled the ROME-IV criteria^3^ for IBS (IBS-C *n* = 133, IBS-M *n* = 237 IBS-D *n* = 246, IBS-U *n* = 26). Further functional bowel disorder diagnoses yielded were functional constipation (*n* = 173) and functional diarrhoea (*n* = 157). The remaining 203 patients demonstrated symptoms rendering them non-classifiable due to syndromic overlap in (*n* = 130) or exclusion from (*n* = 73) current classification systems.

**Fig. 2:**
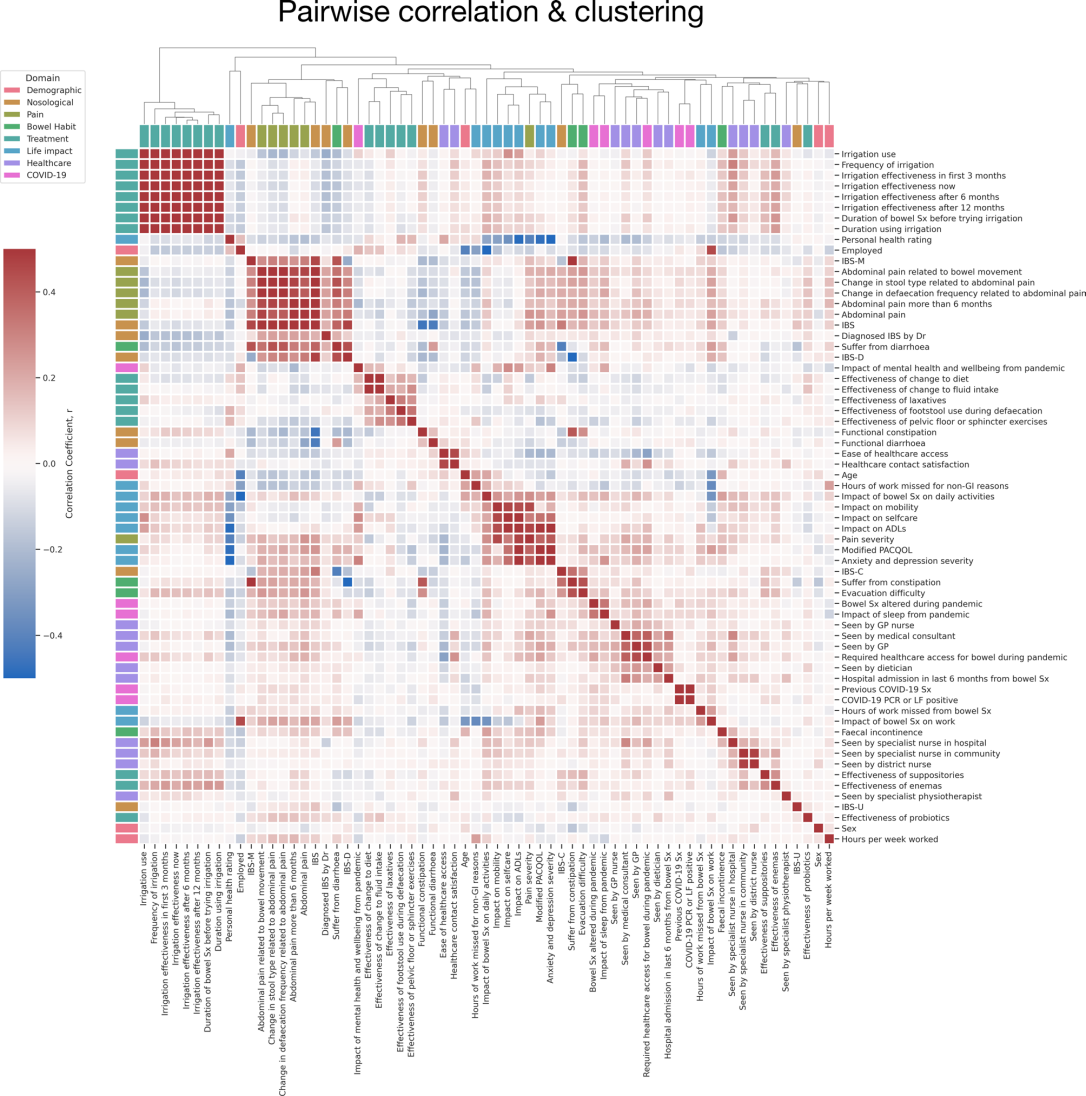
Feature correlation matrix dendrogram. The correlation matrix was derived from the Pearson correlation coefficient, and the hierarchical clustering dendrogram was derived from the Euclidean distance matrix. Darker red squares depict more positive, and darker blue squares depict more negative correlation coefficients between pairwise factors. Abbreviations: ADLs, activities of daily living; GP general practitioner; IBS, irritable bowel syndrome (-C constipation, -D diarrhoea, -M mixed, -U unclassifiable); LF, lateral flow; PCR, polymerase chain reaction; Sx, symptoms.

We derived a hierarchical clustering representation of the interrelationships between patient factors based on pairwise correlation coefficients (Fig. 2). This illustrates that when comparing simplistic linear relationships between patient factors, they generally align with self-explanatory domains. For example, measures of irrigation treatment use and its patient-reported effectiveness were highly correlated and clustered together (*r* ≥ 0.64, all FDR-corrected *p* < 0.0001). Similarly, measures of pain were highly correlated and clustered together, as well as pain-criterion diagnoses such as IBS (*r* ≥ 0.40, all FDR-corrected *p* < 0.0001). Patient-reported effectiveness of several treatments formed another cluster, both medicinal (laxative use) and non-medicinal (including changes to diet, fluid intake, footstool use, and pelvic or sphincter exercises) (r range 0.13–0.50, all FDR-corrected *p* < 0.0001). Pain severity, the impact of bowel symptoms on daily activities, and the impact on measures of assisted daily living (ADLs) formed another cluster (*r* ≥ 0.32, all FDR-corrected *p* < 0.0001). Finally, engagement and the requirement of healthcare services formed a weak cluster, including visits to a medical consultant, general practitioner, dietician, and nurse (r range 0.12–0.45, FDR-corrected *p* < 0.0001).

Taken together, these initial analyses show that many aspects of patient data cluster together into relatively self-explanatory domains when modelled in simple pairwise linear terms. Measures of abdominal pain (and FBD diagnoses made by the presence of pain^3^ cluster together. Where an aspect of a patient’s daily life is disrupted, disruption to other aspects of their life is also likely to occur. Where a response to one treatment is identified, there is likely to be some response to another. Key here, however, is that such an approach only superficially characterises pairwise and linear relationships between a patient or disease factor; a remit nonlinear machine models allow us to interrogate further.

### Machine model predictions of all patient factors

**Fig. 3:**
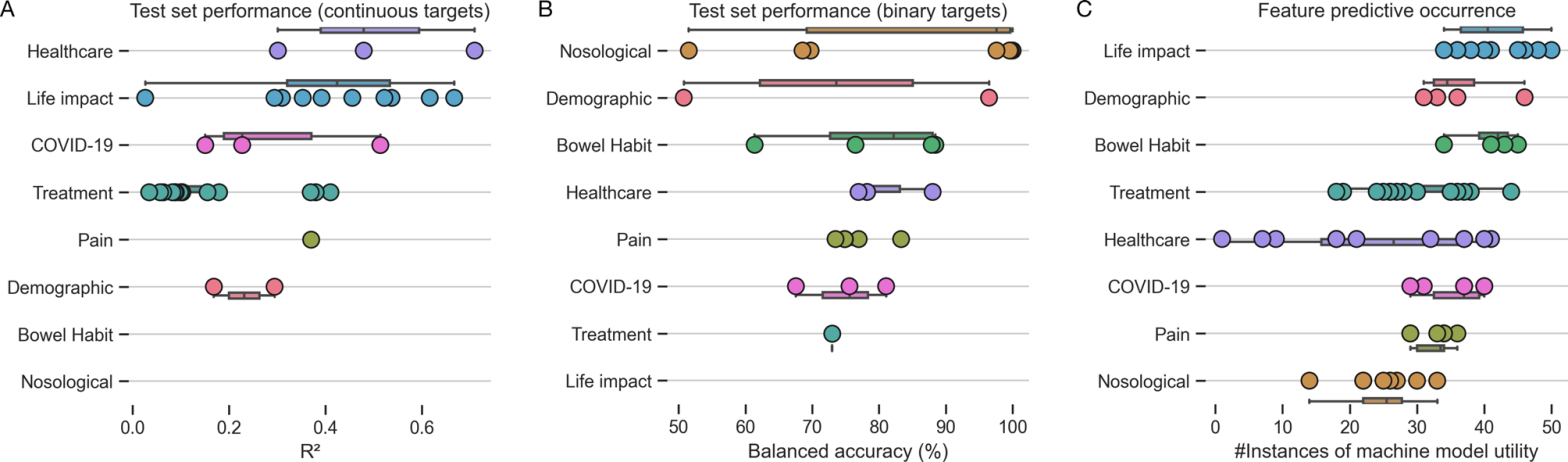
Machine model performances and feature importance across all domains. **A**) Test-set performance for regression models (in R^2^. **B**) Test-set performance for classification models (in % balanced accuracy). **C**) Feature occurrence across all modelling tasks, illustrating the frequent use of life impact measures in machine model predictions, where diagnostic (nosological) data use was uncommon. All panels are stratified and colour-coded by data domain, as shown on the y-axis.

The out-of-sample test set performance breakdowns for all models across the different data domains are shown in Fig. 3; Table 1, and Table 2 and described in greater detail within the supplementary material (Supplementary Fig. 1). Regression models (Fig. 3A; Table 1) tasked to predict healthcare usage and life impact achieved the best out-of-sample predictive performances, with more variable performances across treatment, pain, demographic, and COVID-19 impact domains. These findings illustrate how the healthcare requirements of a given individual could be relatively well predicted with machine learning, plausibly applicable to triage systems or healthcare system planning, and similarly how the impact on daily life was relatively easily predicted also, relevant to the broader impact of disease at both the individual and societal level. In contrast, predicting individual treatment response in this cohort was a far more challenging task.

The classification models in panel Fig. 3B; Table 2 demonstrate that disease classification (nosology) was overall most predictable, followed by bowel habit, healthcare usage, pain, COVID-19 impact, and treatment data. The high classification accuracy of patient diagnosis is an expected finding, given the clear constellation of signs and symptoms that algorithmically determine them^4^. Yet, the ability to accurately elucidate employment status, healthcare usage (and type of) at the individual level holds plausible value for quantifying the wider impacts associated with FBDs, pertinent to the patient’s lived experience.

**Table 1:** Out-of-sample test set performances for regression models. Model performance is given by the R^2^ value, where a higher value indicates greater predictability from the remaining patient data. Abbreviations: adls, activities of daily living; PACQOL, patient assessment of constipation-related quality of life.

Regression model target	Test set performance (*R*^2^)
Frequency of attendance for bowel symptoms	0.71
Impact of bowel symptoms on daily activities	0.67
Impact of bowel symptoms on work	0.62
Anxiety and depression severity	0.54
Impact on ADLs	0.52
Impact of mental health and wellbeing from the pandemic	0.51
Ease of healthcare access	0.48
Modified PACQOL	0.46
Effectiveness of pelvic floor or sphincter exercises	0.41
Impact on selfcare	0.39
Effectiveness of fluid intake	0.38
Pain severity	0.37
Effectiveness of change to diet	0.37
Personal health rating	0.35
Hours of work missed for non-GI reasons	0.31
Healthcare contact satisfaction	0.30
Age	0.29
Impact on mobility	0.29
Impact of sleep from the pandemic	0.23
Effectiveness of footstool use during defecation	0.18
Hours per week worked	0.17
Effectiveness of laxatives	0.16
Bowel symptoms during the pandemic	0.15
Effectiveness of probiotics	0.10
Effectiveness of enemas	0.09
Effectiveness of suppositories	0.08

**Table 2:** Out-of-sample test set performances for classification models. Model performance is given by percentage balanced accuracy, where a higher value indicates greater predictability from the remaining patient data.

Classification model target	Test set performance (balanced accuracy %)
Diagnosis of IBS	100%
Employment status	96%
If suffering from diarrhea or constipation	88%
If seen by a GP for FBD	88%
If suffering from abdominal pain	83%
If required healthcare access for bowel reasons during the pandemic	81%
If seen by a specialist hospital nurse	78%
Presence of abdominal pain for six months or more	77%
If seen by a medical consultant for FBD	77%
If suffering with evacuation difficulty	76%
Change in stool types related to abdominal pain	75%
Abdominal pain related to bowel movements	73%
Irrigation use	73%
If diagnosed as IBS by a doctor specifically	70%
Diagnosis - functional constipation	69%
Diagnosis - fecal incontinence	61%
Diagnosis - functional diarrhea	52%
Sex	51%

A key advantage of machine learning is its ability to undertake feature selection, automatically choosing the strongest predictors of a given modelling target to build the best-performing model. In reviewing the feature importance and contributions across all model targets, it transpired that whilst patient factors of life impact, demographics, and bowel habit were commonly selected by XGBoost runs, rarely was the patient’s diagnostic label selected, suggesting that diagnosis was, in fact, minimally helpful for a model to predict wider patient factors, including those of their lived experience (Fig. 3C).

### Associations of symptom burden and quality of life

**Fig. 4:**
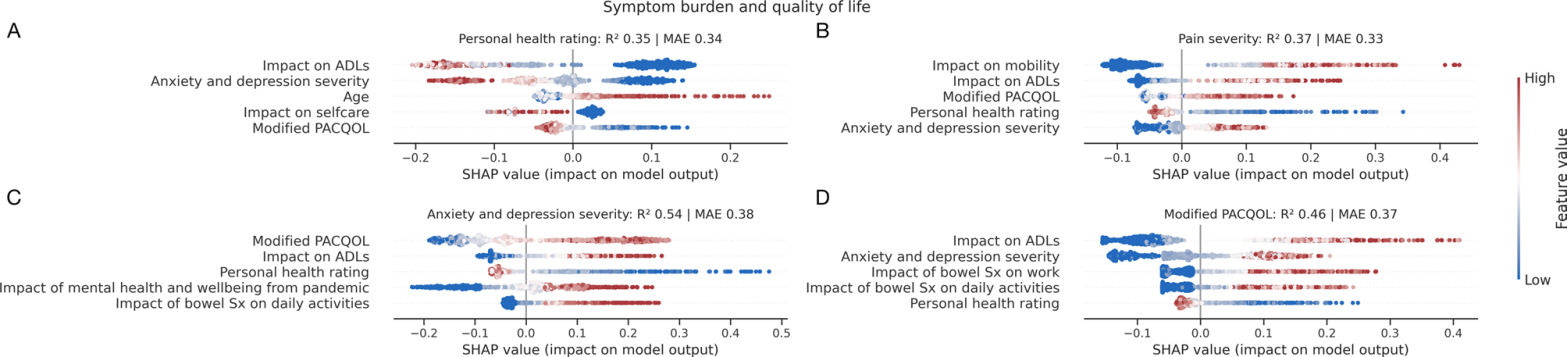
Associations of symptom burden and quality of life. SHAP plots for machine learning models quantifying patient **A**) personal health rating, **B**) pain severity, **C**) anxiety and depression severity, and **D**) modified PACQOL. Out-of-sample performance is shown by R^2^ and mean absolute error (MAE). Only the top 5 predictive factors of each target are shown for visualisation purposes. For each panel, each point represents a patient, and each row is an input feature to the model, where positive x-axis values depict a *positive* impact on the model output, and redder points depict higher feature values. For example, panel **A**) shows the top predictive feature for personal health rating to impact on ADLs, where a greater (i.e., more detrimental) impact on ADLs was associated with the patient reporting a lower (i.e., worse) personal health rating. Key here is that patient diagnosis was not selected by the models as informative in their prediction. Abbreviations: ADL, activities of daily living; PACQOL, patient assessment of constipation-quality of life; Sx, symptoms.

Models predicted symptom burden and quality of life measures relatively well, relying predominantly on life impact, mental well-being, and age to inform them, but notably, not diagnostic labels. We provide a breakdown of performant models in predicting symptom burden and quality of life metrics with SHAP plots in Fig. 4, further discussed in the supplementary material.

### Associations of life impact

**Fig. 5:**
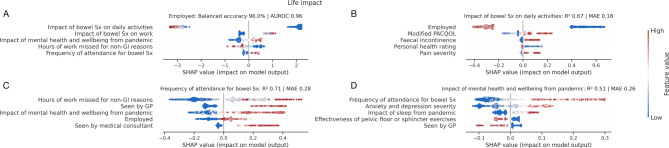
Associations of life impact. SHAP plots for machine learning models quantifying patient **A**) employment status, **B**) impact of bowel symptoms on daily activities, **C**) frequency of healthcare attendance for bowel symptoms, and **D**) impact of mental health and wellbeing from the pandemic. Out-of-sample performance is shown by % balanced accuracy and AUROC for classification models (**A**), with R^2^ and mean absolute error (MAE) for regression models (**B**-**D**). Only the top 5 predictive factors of each target are shown for visualisation purposes. A description of interpreting SHAP plots is given in the legend in Fig. 4. Patient diagnosis was not selected by the models as informative in their prediction. Abbreviations: GI, gastrointestinal; GP, general practitioner; PACQOL, patient assessment of constipation-quality of life; Sx, symptoms.

Models accurately predicted life impact targets, primarily informed by hospital attendance data, employment status, other life impact measures, mental well-being, and pain data, but notably not diagnostic labels. In Fig. 5, we provide a breakdown of performant models in predicting life impact with SHAP plots.

### Associations of patient-reported treatment effectiveness

**Fig. 6:**
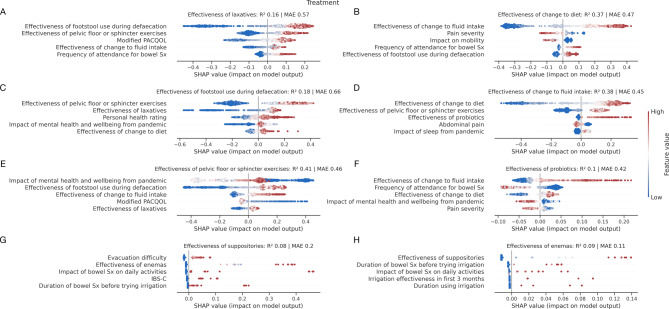
Associations of treatment response. SHAP plots for machine learning models predicting the effectiveness of **A**) laxatives, **B**) dietary changes, **C**) footstool usage during defecation, **D**) fluid intake changes, **E**) pelvic floor or sphincter exercises, **F**) probiotics, **G**) suppositories, and **H**) enemas. Out-of-sample performance is shown by R^2^ and mean absolute error (MAE). Only the top 5 predictive factors of each target are shown for visualisation purposes. A description of interpreting SHAP plots is given in the legend in Fig. 4. Diagnosis only features in one of eight treatment models, where predictive performance was also notably poor (panel **G**). Abbreviations: IBS, irritable-bowel syndrome; PACQOL, patient assessment of constipation-quality of life; Sx, symptoms.

Model performance in predicting patient-perceived treatment effectiveness was more variable. Despite variable performance, for which some caution should be taken in its interpretation, patient-reported treatment response to one intervention was largely predictive of response to another. Conversely, those refractory to one intervention were likely to be refractory to others. In Fig. 6, we provide a breakdown of performant models in predicting patient-perceived treatment effectiveness with SHAP plots.

The foregoing analyses illuminate relationships between singular aspects of the patient’s lived experience. While disclosing non-linear and higher-order relationships between sets of factors in predicting another, the approach lacks an all-encompassing compact summary of the patient’s lived experience that only an unsupervised approach could plausibly offer. Given the interweaving of disease and patient features, this may be best approximated by a generative model of a network^20,21^.

**Fig. 7:**
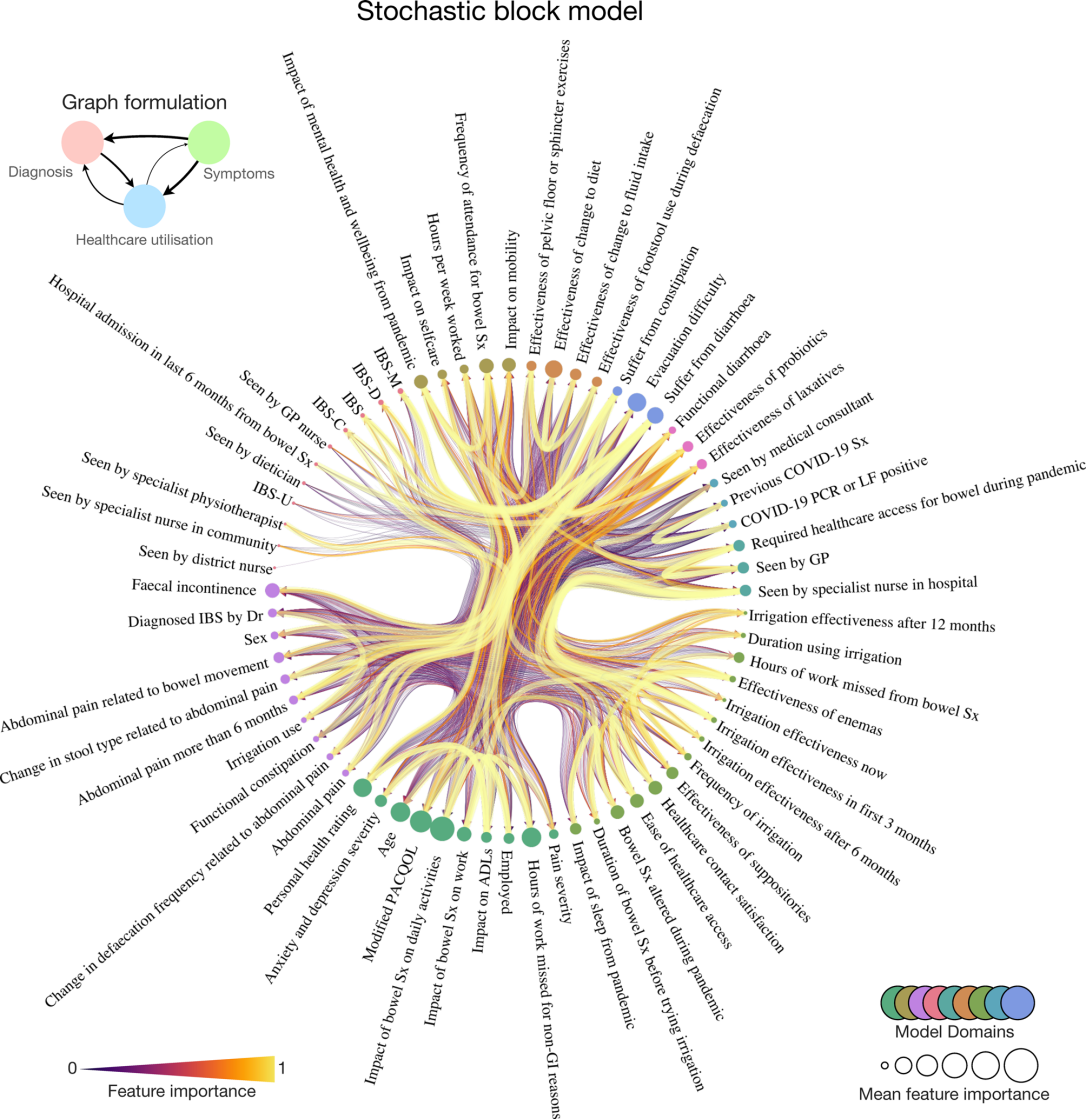
A generative network community structure for the lived experience of functional bowel disorders. Radial network of the nested, generative Bayesian stochastic block model community structure of patient factors. Nodes are individual circles with corresponding text labels, sized according to their importance in the statistical prediction of all other target nodes. Edges are weighted by the directional feature importance in predicting one feature over another, where edge width and colour are proportional to the key. Node communities are similarly colour-coded as per the key at the second hierarchical level. Abbreviations: ADLs, activities of daily living; GP general practitioner; IBS, irritable bowel syndrome (-C constipation, -D diarrhoea, -M mixed, -U unclassifiable); LF, lateral flow; PCR, polymerase chain reaction; Sx, symptoms.

We therefore fitted a nested generative stochastic block model^22^ comprising all factors as nodes, with weighted directed edges as feature contributions to each machine model, revealing a sophisticated community structure of patient factors (Fig. 7, Supplementary Fig. 2). This was broadly organised into the domains of nosology, life impact, treatment effects, and symptomology. The network structure reiterated the importance of symptom and life-impact factors instead of diagnosis-related ones.

Next, we extracted each community block’s weighted eigenvector, hub, authority, and centrality metrics at the second nested (L_1_) level. Eigenvector centrality measures a node’s ‘influence’ across the whole network^23^. The Hyperlink-Induced Topic Search (HITS) is a centrality algorithm historically developed for rating worldwide web pages, stemming from the observation that when the internet was initially forming, specific web pages operated as large directories – hubs – yet were not authoritative to the information contained within them, although were indeed helpful as catalogues to direct people to the authoritative pages^24,25^. Framed differently, a ‘good hub’ of a network of the internet points to many other pages, whilst a ‘good authority’ would be a page linked by many different hubs^26^.

**Fig. 8:**
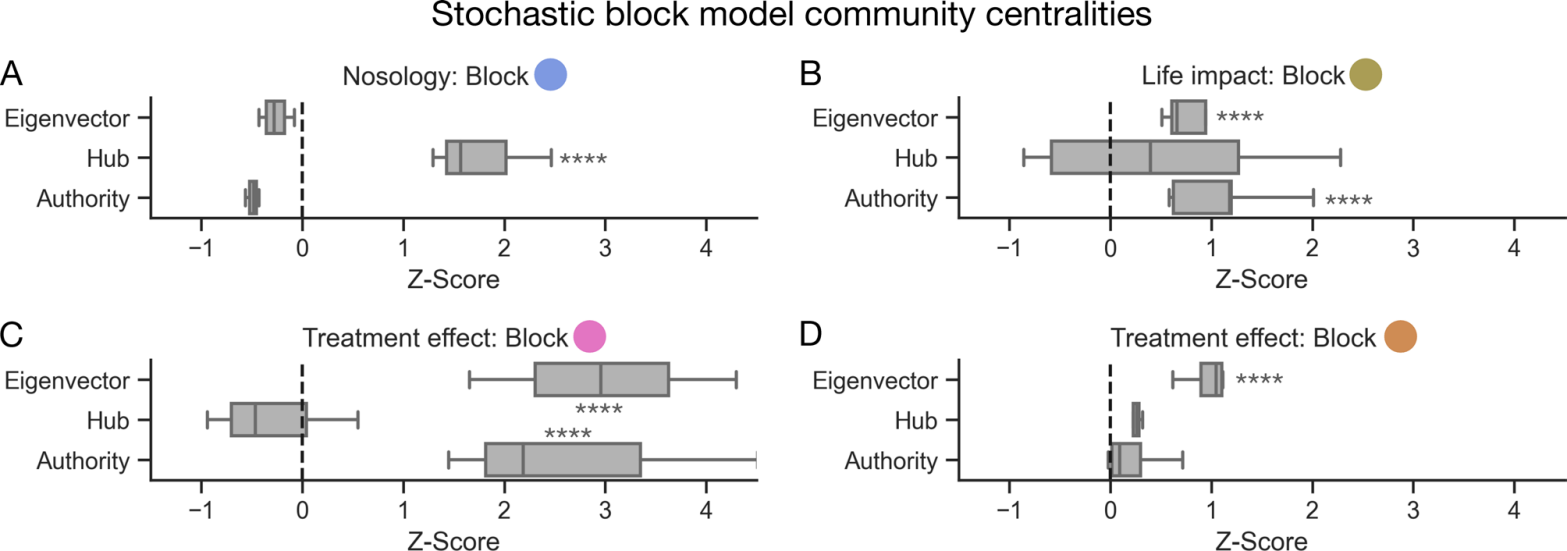
Diagnoses are hubs, but life impacts and treatment effects are authorities and influencers. Box and whisker plots illustrating centrality metrics of **A**) a nosology node community from the nested stochastic block model comprising if the patient suffers from diarrhoea, constipation, or any form of evacuatory difficulty; **B**) life-impact including self-care, mobility, ability to work, healthcare access and mental wellbeing; **C**) the treatment effectivity node community comprising the effects of probiotics, effects of laxatives, and also a diagnosis of functional diarrhoea; and **D**) the treatment effectivity node community comprising the effects of pelvic floor/sphincter exercises, dietary or fluid changes, and footstool use during defecation. Refer to Fig. 7 for a radial representation of this network community structure. **** denotes a post-hoc Tukey significance test of *p* < 0.0001 following one-way ANOVA.

This quantitative analysis of our generative network structure found the node community consisting of constipation or diarrheal disease nosology had significantly greater hub centrality than all other node blocks (the measure of how often a node links to other factors irrespective of how informative or authoritative it may be) (one-way ANOVA with post-hoc Tukey *p* < 0.0001) (Fig. 8). Meanwhile, two communities consisting of treatment effects and life impact measures had significantly greater eigenvector centrality (the measure of a node’s ‘influence’ across the whole network) (one-way ANOVA with post-hoc Tukey, *p* < 0.0001). Similarly, the node communities related to treatment effectiveness of probiotics and laxatives, as well as the node community related to life impact, yielded significantly greater authority centrality (the measure of how informative and authoritative a node is to the remaining network) (one-way ANOVA with post-hoc Tukey, *p* < 0.0001).

## Discussion

Modern medicine has become an algorithmic science: the clinician assesses symptoms, orders tests, and classifies the illness into a diagnostic category on which therapy is based^3,27^. In FBDs, there are no objective measures that sub-classify patients based on pathophysiology; instead, symptom-based classifications are crafted to aid management. Whilst this can be helpful, it may oversimplify the portrayal of a complex, multi-dimensional condition, where linkage to lived experience is vital^1,2^. We have conducted a holistic characterisation of a large cohort of patients with FBD using a multi-dimensional machine learning approach without prior assumptions on the associations of patient lived experience. The main pertinent findings are twofold:

Firstly, we reveal in unprecedented detail the associations of patient-reported symptom burden, quality of life, life impact, and treatment effectiveness and provide the framework for predicting them. In many ways, these are at odds with what we, as healthcare professionals, often assign as the major impacts of health and wellbeing; examples are discussed below.

Secondly, our generative network analysis, summarising the output of a comprehensive machine modelling framework, reveals the high-dimensional community structure of these patient factors. This process formally quantifies that whilst the nosological domains of disease we classify patients with are network hubs that link many aspects of patient health and wellbeing, they are poorly influential (or authoritative) in describing the broader aspects of patient health. Instead, patient-reported treatment effectiveness and impact on a patient’s daily life are quantitatively authoritative and influential.

The value in developing a suite of machine models to predict these characteristics is not merely the depiction of those with high performance (which is most often the case in machine learning and digital medicine research). Rather, this process illuminates what can (but equally importantly, what cannot) be predicted from data routinely available in a clinical setting. It should come as no surprise that a diagnosis of IBS can be predicted perfectly from metrics of abdominal pain and gastrointestinal disturbance: these factors are definitional for a diagnosis of IBS by current classification systems^4,6^. More important, however, is the fidelity of models in ascertaining other aspects of patient health, such as the effects of young age or employment status, which are not as intuitive.

Machine learning models are often described as sophisticated in their ability to formulate a decision based on nonlinear interactions amongst complex multivariate data^16,28^but the reality is that the healthcare professional reviewing a patient undertakes similar processes to inform their clinical decision-making^16^. To that end—and with a priority of improving digital health for these individuals—we have identified key factors in a machine learning model’s prediction of ill health from the individual patient’s perspective, so that these factors can be considered during clinical consultations.

Here, we find that a model predicting a patient’s health rating is not principally predicted by the severity of gastrointestinal symptoms or diagnosis, but instead by the impact of disease on their daily life, the presence of anxiety and depression, and being ill at a younger age. While most of these factors are intuitive and unsurprising, the effect of young age on the perception of well-being has not been previously highlighted.

The greatest modelling associations of patient-reported pain severity were the impact of illness on daily life activities, the presence of anxiety and depression, and the patient’s personal health rating. Quality of life predictions were best predicted by the impact of the illness on daily activities, the presence of anxiety and depression, and the impact of bowel symptoms on work. These results formalize the importance of considering not just the gastrointestinal symptoms of FBDs, but rather the impact they exert on patient life.

Other than in suppository use for if there was a diagnosis of IBS-C (a trivial association), the diagnostic label that we as healthcare professionals assign to these patients as part of ‘best practice’ did not feature as a ‘top five feature’ for any machine model. Indeed, none of the top five features of a patient’s health rating specifically interrogated gastrointestinal symptoms, emphasizing the importance of quantifying the impact of life factors (such as employment and daily life) and mental well-being throughout their routine clinical care.

Turning to measures of life impact, patient employment status could be predicted by a machine learning model (the model correctly predicted employment status in 229 of 235 out-of-sample test cases). The greatest modelling predictor of employment status was, by some distance, the degree of impact of bowel symptoms on daily activity, for which an association seems plausible. Conversely, the greatest predictor of patient-reported impact of bowel symptoms on daily activities was unemployment. Regardless of causal directionality, it seems reasonable to suggest that simply ascertaining employment status is a strong indicator of general life impact measures and something that should be considered in every consultation.

One of the highest-performing machine models was in delineating the frequency of patient healthcare attendance for bowel symptoms (which achieved an out-of-sample R^2^ of 0.71). The greatest model predictors of healthcare attendance were the hours of work missed, whether a GP had already seen the patient, the impact on their mental health, employment status, and whether they had seen a medical consultant before. Vitally, the patient frequency of healthcare attendance for bowel symptoms was not predicted by bowel symptomology, but instead a combination of healthcare access/gatekeeping (i.e., if already known to the service), impact on employment and life, and mental wellbeing. This is in keeping with the previously known evidence of healthcare-seeking in IBS^29,30^. Naturally, quantifying healthcare requirements is essential, as it has implications in designing, planning, and budgeting healthcare service provisions^31^.

Lastly, we briefly draw focus to our model predicting the impact of mental health and well-being during the COVID-19 pandemic. The strongest predictor in this cohort was the frequency of attendance for bowel symptoms. Put another way, mental health in this FBD population was most associated with the ability to access healthcare during a time when the health service was under particular strain. This finding conveys the priority we should place on widening and maximising patient healthcare access to safeguard mental well-being.

Several models in this analysis aimed to quantify patient-reported effectiveness of routinely provided FBD treatments, both medicinal (laxatives, enemas, suppositories, probiotics) and non-medicinal (dietary or fluid changes, footstool use during defecation, and pelvic floor/sphincter exercises). We would be cautious about drawing conclusions when comparing the effectiveness of certain treatments to others, as the study was not designed as a clinical trial to facilitate this comparison. However, the critical insight was in the modelling predictors of patient-reported treatment effectiveness. Namely, the greatest predictor of a patient’s response to *any* treatment was their response to *any other* treatment. Once again, symptomatology and disease classification were minimally predictive of treatment response, but other factors (social, psychological, and comorbid) combined to create, in some patients, a state of refractoriness. It is these patients who respond poorly to all treatments that are seen in secondary and tertiary care and require a holistic approach. More research is needed to understand refractoriness in FBD.

Finally, we harmonised the findings to construct a Bayesian generative network revealing the community structure of factors affecting those living with FBDs. Broadly, these feature communities coalesce to nosology, life impact, treatment effects, and symptomology. Interestingly, however, we show that the nosological branch of these factors – i.e., the domains of disease leading to diagnostic labels we assign to these patients – are ‘hubs’ in this network (a feature that points to many others) but are, in fact, minimally influential^23,24,32^. Instead, the communities of life impact and treatment effectiveness are quantitatively more influential (with higher eigenvector centrality) and authoritative (with higher authority centrality) in the remaining aspects of their health. These findings suggest that the assignment of disease labels to these patients can only help so far in disclosing or influencing the broader FBD lived experience, as shown elsewhere in the comparison of constipation-predominant irritable bowel syndrome and functional constipation^6,33^. Instead, we should focus more on reducing life impacts and improving treatment effects through a holistic approach.

The predictive models and network insights presented here offer multiple pathways for clinical integration. In routine practice, our finding that employment status serves as a powerful predictor of life impact could be operationalised through simple screening questions at initial consultation, triggering more comprehensive assessments when unemployment is identified. The machine learning framework could be deployed as a clinical decision support tool, providing real-time predictions of healthcare utilisation needs, allowing optimised resource allocation and means to identify patients requiring more intensive support^31^. For clinical trial design, our discovery that treatment responders cluster together while non-responders remain refractory across interventions has immediate implications. Future trials could stratify patients based on their predicted treatment response probability, potentially using a composite score derived from previous treatment responses, employment status, and life impact measures. Such an approach may enable more efficient trial designs, smaller sample sizes for detecting treatment effects in responsive subgroups, and the development of targeted interventions for the refractory population. Electronic health record integration could automate much of this stratification, utilising the demonstrated predictive features to identify suitable trial patients more effectively prospectively, in line with other trial applications using deep modelling^34^.

Our findings fundamentally challenge the primacy of diagnostic classification in critical aspects of FBD management. Clinically, such a paradigm shift would ultimately require a change in medical dogma to prioritise holistic assessment over diagnostic labelling, but this is certainly not the first evidence to support it^1,6,35,36^. It would seem rational for professional bodies to revise their reliance on specific disease labels for clinical guidelines in these settings, moving from diagnosis-driven algorithms to patient-centred frameworks. Regulatory implications are equally profound. Current drug approval pathways rely heavily on disease-specific indications and diagnostic criteria for patient selection, but if a given diagnosis is either uncertain or minimally informative to a patient’s actual experience, the knowledge that can be drawn from such an approach will always be limited. Our finding that diagnosis poorly predicts treatment response suggests evaluating interventions based on multidimensional patient phenotypes rather than traditional disease categories. This could accelerate personalised medicine approaches, but would require significant innovation towards new evidence standards that only large-scale data, statistical rigour, and cross-disciplinary collaboration could provide^18,37,38^. Although this may all sound controversial, it in reality closely aligns with what William Osler wrote now more than 125 years ago: “*Care more particularly for the individual patient than for the especial features of the disease”*^39^.

The study quantified a breadth of patient factors feasibly acquired during routine clinical care, ranging from demographics, diagnostic information, symptomatology, quality of life, healthcare access, and patient-reported effectiveness of regularly administered treatments. One limitation is that we did not quantify more comprehensive and/or specialist investigations (e.g., microbiota, gastrointestinal imaging, genetic, or an exhaustive list of comorbidity data) since this would have limited the applicability and generalizability of the findings to centres in which these are not part of routine care. We could not control for an individual’s consent to participate in the study or their individual response rate; thus, selection bias is possible. To our strength, in maximising the quantification of variables that *are* routinely available in a real-world healthcare setting, we were able to sample a large cohort from which we could construct a suite of machine learning models with proven fidelity that could be evaluated and/or deployed in similar centres in further research. Whilst our study covers a substantially larger UK geospatial footprint and referral site catchment than most others of its kind, and its clinicodemographic distribution closely follows the broader epidemiology of these disorders, additional external validation with broader representation would strengthen findings further and should form a key step before clinical adoption.

Secondly, the study was not designed to trial specific treatments. Instead, it was designed as a cross-sectional study, where patients could self-report the effectiveness of the therapies they had experienced throughout their care. This limits inference that could instead be drawn from an experimental allocation, i.e., a randomised controlled trial, but it does quantify the response to treatment with explicit emphasis on the individual patient experience (as opposed to any biochemical/investigatory endpoint). In any case, our focus was to illuminate the associations between patient-reported responses to treatment *in general*, rather than quantifying the superiority or non-inferiority of one treatment over another.

Thirdly, our study was not designed to investigate directional effects, nor exhaustive statistical inference beyond reasonable computing power. One might suggest a plausible directionality of impaired GI health leading to a triad of increased healthcare utilisation, loss of productivity/employment, and worsening QoL/mental well-being, but it must conform to the appropriate criteria for establishing causality^40^. An additional analytical route would be dedicated causal inference, a task for future research^41^. Furthermore, alternative statistical approaches such as multiple imputation datasets/nested cross-validation tasks would offer additional opportunity for statistical testing. However, this was not undertaken here for in supplementary experimentation we found it carried little to no effect.

In conclusion, we demonstrate a machine learning and Bayesian generative network approach for deep phenotyping patient disease and lived experience, first applied to characterise FBDs. Our hypothesis-generating framework reveals new insights into the model predictors of patient-reported symptom burden, quality of life, impact on daily life, and treatment effectiveness in a large representative cohort. Strikingly, these predictors are often at odds with what we, as healthcare professionals, typically presuppose are the most important. Instead of disease classification or symptom severity, this was best predicted by the impact on daily life, employment status, access to healthcare, and mental well-being. The modelling predictors of an individual’s mental well-being were their access to healthcare. Although not definitively causal, we urge that healthcare access be maximised and that a holistic approach to consultation is required for these individuals. Patients tend to be responsive to multiple therapies or refractory to all, and a deeper understanding of refractoriness should form a future research priority. In demonstrating the application of our framework to FBDs here, we hope to catalyse further study of the patient’s lived experience across broader health remits.

## Methods

### Ethical approval

The study was approved by the local institutional review board and conducted in accordance with the Declaration of Helsinki. The Health Research Authority approved this study prior to commencement. REC reference 21/SW/0086 (IRAS ID 296856). Informed consent was obtained from all participants.

### Study design

A single online questionnaire was administered to two existing cohorts of individuals using convenience sampling methods. The two groups were:

1. ContactMe-IBS (established 2017) – a national irritable bowel syndrome (IBS) registry of people who are interested in participating in IBS research (https://www.contactme-ibs.co.uk/). ContactMe-IBS is owned by the NHS (County Durham and Darlington NHS Trust). Registrants are primarily from the Northeast (actively promoted within Durham Bowel Dysfunction Service) and the Southwest, where GPs are particularly research active with ContactMe-IBS. Access to the registry is available via numerous sources, including GP practices, gastroenterology clinics, pharmacies, and social media. During registration, participants self-identify as having IBS by completing screening questions based on Rome IV criteria^4^.
2. Transanal irrigation (TAI) database (established 2019) – a database of patients who have commenced TAI under the care of Durham Bowel Dysfunction Service.

Participants on the registry received primary or secondary care for IBS and gave permission to be informed of active research studies. Over four weeks, October - November 2021, registrants of both databases (*n* = 4480 on ContactMe-IBS; *n* = 259 on the TAI database) were invited to participate by email link to a questionnaire or postal questionnaire if preferred. Online questionnaire data were captured digitally via the web-based REDCap application, a secure system designed to support data collection for research studies. Inclusion in the study required participants to be 18 years or older with symptoms of bowel dysfunction, registered on either database and able to understand written and spoken English (for questionnaire completion). Participants who did not respond to the invitation or reminder email or those who did not fully complete the questionnaire were excluded.

### Materials

The study used an 88-item questionnaire requiring ~ 35 min to complete, organised in the following sections:

1. *Demographic*: including date of birth, sex, ethnicity, and employment status.
2. *Nosological*: this section was designed to characterise the FBD type of the participant. The scoring algorithms of the ROME IV^4^ criteria were used to identify primary diagnostic groups: irritable bowel syndrome (constipation [IBS-C], diarrhoea [IBS-D] predominant, or mixed [IBS-M]); functional constipation (FC); functional diarrhoea (FD); or faecal incontinence (FI). Criteria for evacuatory dysfunction (ED) did not depend on investigations but relied on symptom scores for straining, a feeling of blockage, a feeling of incomplete evacuation and the need to digitate, with questions and scoring of these aligned to the ROME IV questionnaire.
3. *Primary symptom*: respondents were asked to report their primary symptom from a choice of ‘abdominal pain’, ‘bloating’, ‘watery stools’, ‘hard stools’, and ‘frequent bowel movements’, including quantified severity and duration experienced.
4. *Bowel habit*: the Bristol Stool Form Scale was used to identify stool type^42^. The ROME IV criteria individual question data was used to assess bowel habit^4^.
5. *Treatment*: A visual analogue scale (VAS) was used to measure perceived effectiveness for a range of trialled treatments. These included medicinal (such as laxative, enema, suppository) and non-medicinal (such as pelvic floor/sphincter exercises, footstool use during defecation, fluid and/or dietary changes). The study team developed questions on the use and effectiveness of TAI for managing FBDs, consisting of seven single-answer multiple-choice questions and a VAS for patient-perceived effectiveness. Data were not curated or designed for treatment comparisons but rather to delineate the model predictors of patient-perceived effectiveness of a given regime.
6. *Life impact*: the impact of FBDs on QoL was assessed using a 5-point Likert scale based on the Patient Assessment of Constipation on Quality of Life (PAC-QOL) questionnaire^8,43^. PAC-QOL wording was widened to reflect all FBDs; for example, ‘constipation’ was amended to ‘bowel symptoms’, and questions related directly to constipation were omitted (Q2, Q4, Q20, Q21, Q24 from PAC-QOL^43^. This approach would enable insight into the impact of any set of bowel symptoms on a patient rather than placing focus on specific disease subtypes. The Eq. 5D-5 L General Health^44^ was used to explore the impact of FBDs (on mobility, self-care, usual activities, pain or discomfort, and anxiety or depression. Each patient’s rating of their overall health was also measured by VAS. The Work Productivity and Activity Impairment Questionnaire^45^ assessed impairment in activities of daily living (ADL) and employment-related productivity. Questions elicited employment status, absenteeism (percentage of work hours missed due to bowel symptoms), presenteeism (the degree to which symptoms affect work productivity whilst working), percentage of work hours missed for other reasons, and the degree to which symptoms affected other ADLs in the preceding seven days.
7. *Healthcare use*: questions determined whether the participant had been admitted to hospital for bowel symptoms; and their access to healthcare including physiotherapy, general practitioner (GP), consultant gastroenterologist, GP/district/specialist nurse, and dietician.
8. *COVID-19*: comprising single response multiple choice questions explored how the COVID-19 pandemic affected individuals.

### Algorithmic approach

FBDs are a complex set of disorders that are both impacted by and have a profound impact on a wide array of interacting biological, social, and psychological factors. A study seeking to model or characterise *one* single constitutional, diagnosis, disease, treatment, life impact, or healthcare access feature in such a cohort could only increase its understanding by small margins. Our task here is to find a means to understand these patients’ disease processes and lived experiences in a much broader sense, developing a suite of statistical models aiming to predict *all* patient factors instead. In undertaking such an approach, we forgo any clinical assumptions, instead harnessing a data-driven method that allows machine models to identify which constitutional, diagnostic, disease, treatment, life impact, or healthcare access factors are predictable, whilst simultaneously revealing their own predictors. Our framework tests the hypotheses that (1) a machine model shall discern what patient factors plausibly can – and perhaps equally important, what cannot be – predicted from their remaining data, and (2) a machine model shall identify the greatest statistical predictors of a given patient feature, both of which have downstream clinical utility in decision support, patient monitoring, and treatment.

A practical example is the prediction of patient-reported health quality. In determining the extent to which patient-reported health quality can be predicted from other constitutional and clinical data, this reveals the capacity for healthcare professionals to ascertain it from data routinely available. Where patient-reported health quality is readily predictable by a machine model, then its associated factor(s) can guide practice, whether for patient triaging or treatment monitoring. If, however, health quality is not predictable, then this informs us that data currently curated, inclusive of the patient constitution, diagnosis, and healthcare access, do not inform it, so in our practice, we should not make assumptions as to how a patient would rate their personal health without seeking further information.

In line with standard practices, we firstly removed patients with missing data for > 40% of feature columns (*n* = 1) and conversely removed features with missing data for > 40% of patients (f = 19), resulting in 59 features. We established that the data was missing at random (MAR) and used multivariate imputation via chained equations (MICE)^46^ with predictive mean matching, 50 multiple imputations and 50 iterations to impute the remaining missing values. These parameters were tenfold the default for each, at the expense of a significant computational burden to maximise result validity. This process was undertaken before any downstream predictive modelling task. We benchmarked the validity and stability of this imputation process across multiple draws, showing high reproducibility (Supplementary Fig. 3). Following imputation, the database was locked from any further amendments.

We next created a visualisation of the feature space across all patient domains of demographic, nosological, pain, bowel habit, treatment, life impact, healthcare, and COVID-19 in the form of a cluster map. We computed the pairwise Pearson correlation matrix of all features and performed hierarchical clustering by Euclidean distance metrics to produce a dendrogram^25,47–49^. P values were adjusted by False Discovery Rate^50^. For cross-group comparisons, including patient-reported treatment effectivity, we conducted one-way ANOVAs with post-hoc Tukey testing.

We partitioned data 80:20 into model training (*n* = 940) and testing (*n* = 235) sets; the latter was completely excluded through all model development and evaluated only after completing the development of all models. All hyperparameter optimisation was performed using cross-validation only in the training set. We removed features directly related to the target with regular expression to avoid trivially reductive predictions. For example, if the target involved irrigation, all predictive features involving irrigation were dropped. We retained features explicitly related to a given diagnosis (e.g., the presence of abdominal pain when predicting IBS) to be used both to benchmark models, ensuring appropriate fidelity with the modelling architectures and hyperparameters^16,28^and furthermore as vital additional data in the downstream unsupervised modelling task. Targets where the class imbalance exceeded 20:1 were excluded entirely due to insufficient data support. For those remaining, we used Synthetic Minority Over-sampling (SMOTE) to handle class imbalances^51^. This well-established technique oversamples the minority class by creating new cases over a learnt linear manifold^51^. All data were clamped along the 0.1th and 99.9th percentile and normalised to limit the influence of extreme outliers and scale variance, in line with standard practices. SMOTE is applied only to the training set after the fixed train/test split, within which 5-fold cross-validation is performed, ensuring that there is no possibility of data leakage from the test set. Feature normalisation is fitted only on training data and then applied to test data, following standard statistical practice.

For all predictive modelling, we used eXtreme Gradient Boosting (XGBoost)^52^an architecture that employs a parallel ensemble of gradient-boosted weak-learner decision trees. This architecture has shown superior performance in multiple machine learning tasks^52^. Models were constructed as classifiers for categorical targets and regressors for continuous targets. All training runs of a single XGBoost model (for one given target, e.g., patient sex) were undertaken with 5-fold cross-validation within the training data partition. Hyperparameters were optimised by Grid Search across learning rate, number of estimators, maximum depth, subsampling, gamma, and the minimum sum of the instance weight (inside the 80% training data partition), using negative log loss for classifiers and root mean squared error for regressors. This required 16,200 individual model fits per target, which across all 59 targets equated to 955,800 model fits. After identifying the optimum fit, we evaluated model performance on the test set for classifiers, deriving the balanced accuracy, precision, recall, and F1 (all macro-averaged) and for regressors, deriving the mean absolute error, mean squared error, RMSE, and R^2^. MAE and MSE were reported as a function of normalised and z-scored targets to facilitate performance comparison across targets with varying ranges (e.g., the age range was 20–85 years, whereas personal health rating was between 6 and 100).

We derived feature importance scores for all model runs by the number of times a feature is used to split the data across all trees (i.e., the XGBoost default^52^ and feature directionality with SHapley Additive exPlanations (SHAP)^53^in order to shed light on the strongest statistical predictors of each feature and model performance metrics, all evaluated out of sample.

The preceding analyses characterise feature relationships criterion on predicting a singular task: the sex of a patient, their quality of life, burden of disease, *et cetera*. Whilst innovative in disclosing the non-linear and interacting relationships between sets of features in predicting another, the approach lacks an all-encompassing compact summary that only an unsupervised approach could offer. To that end, we turned to graph theory as our solution. Graph theory provides a powerful method of modelling complex systems that combines flexibility with intelligibility^20–22,24,54,55^. It treats individual factors of interest as the “nodes” of a network and their interactions as the connections, or “edges”, between them. Here, nodes were all patient features, and edges were the feature importance indices from XGBoost predictive models, the scaled importance metric of one feature in predicting another. The value of this approach over simple metrics of pairwise similarity (e.g., correlation) is that each edge between features captures the importance of that feature in predicting another. This definitionally incorporates the impact of other features in the graph to yield a high-dimensional community structure whilst formally implementing Occam’s razor by Bayesian inference^21^. We consolidated these findings formally with a nested stochastic block model (SBM), a Bayesian generative model of a network that aims to find the optimal community structure^20,22,47,54–57^. Just as the London Underground network comprises stations (nodes) and tracks between them (edges), organised by different train lines, we study patient factors as nodes and the prediction importance metrics as edges connecting factors to one another. Fitting an SBM to these data reveals the most compact representation of how patient factors are organised, grounded in information theory and Ockham’s razor^56^. We further quantify graph centrality metrics aligned to each community partition to study these organisations of disease and lived experience factors even deeper.

A stochastic block model (SBM)^58^ is a generative model of the community structure of a graph composed of $\:N$ nodes, divided into $\:B$ blocks with edges $\:{e}_{rs}$ between blocks $\:r$ and $\:s$. The model can be framed hierarchically, where edge counts $\:{e}_{rs}$ form block multigraphs with nodes corresponding to individual blocks and edge counts arising as edge multiplicities between block pairs, including self-loops. We seek to infer the most plausible partition $\:\left({b}_{i}\right)\:$of the nodes, where $\:\left({b}_{i}\right)\in\:\:{[1,B]}^{N}$ identifies the block membership of node $\:i$ in observed network $\:G$, with maximisation of the posterior likelihood $\:P\left(G\right|\left({b}_{i}\right))$. The result is a hierarchically organised community structure of nodes assigned into blocks that yields the most compact representation of the graph, as indexed by its minimum description length^56^$\:\sum\:$. The general approach is described in further detail elsewhere^58^. Directed feature importance weights were modelled as exponential. Having initialised a fit, we used simulated annealing to optimise it, with a default inverse temperature of 1 to 10. We did not specify a finite number of draws; rather, we specified a wait step of 1000 iterations for a record-breaking event to ensure that equilibration was driven by changes in the entropy criterion instead of driven by a finite number of iterations as per^20,47,57^.

Having derived a community structure of patient features, we extracted block partitions and derived weighted centrality metrics. These centrality metrics were: (i) eigenvector (a measure of node ‘*influence’* on the overall graph), (ii) authority centrality (a measure of node *authority* in information to other nodes), and (iii) hub centrality (a measure of the propensity for a node to link many other nodes). The full mathematical derivation of these metrics is beyond the scope of this article but well established and discussed in significantly further detail elsewhere^20,23,24,32,47,59^. Our statistical comparison was conducted at the block/community level rather than individual nodes. This aggregation approach partially addresses the independence concern by comparing functionally distinct modules. Prior to statistical testing, all centrality measures were z-score normalised, which helps standardise the distributions and reduce the impact of network-specific biases. We used Tukey’s HSD test rather than simple ANOVA, which provides more conservative multiple comparison corrections that partially account for the increased Type I error risk associated with dependency structures.

## Supplementary Information

Below is the link to the electronic supplementary material.

Supplementary Material 1

## Data Availability

Trained model weights are available upon request from the corresponding author. Data and code availability align with UK government policy on open-source code. Patient data are not available for dissemination under the ethical framework that governs its use.

## Code availability

Patient anonymized code excerpts are available at https://github.com/jamesruffle/perspective-ai. Analyses were predominantly performed within a Python (version 3.6.9) environment with the following software packages: graph-tool^60^, imblearn^61^, Matplotlib^62^, NumPy^63^, pandas^64^, SciPy^49,65^, Scikit-learn^65^, seaborn^48^, SHAP^53^ and XGBoost^52^. MICE was performed in R (version 4.1.3)^46^
