# Supplementary material for: Deep phenotyping of patient lived experience in functional bowel disorders using machine learning
Source: Sci Rep. 2025 Oct 9;15:35349. doi: 10.1038/s41598-025-19273-3 (PMC12511317; doi:10.1038/s41598-025-19273-3)
Supplement: Supplementary file 1 — Supplementary Material 1 [file 41598_2025_19273_MOESM1_ESM.docx]

Supplementary Material

# Results

##

## Supplementary Figure 1: Machine model performances and feature importance heatmaps. Heatmaps of a) regression and b) classification machine models, where the target is shown on the y-axis, and the input feature is shown across the x-axis. In regression models (a), the y-axis target order is based on the out-of-sample R^²^ performance. In contrast, in classification models (b), the order is by out-of-sample balanced accuracy (bracketed values). Input features across the x-axis are coloured by the XGBoost feature importance index, with darker squares indicating features more important in target prediction. The X-axis is sorted by mean-feature importance: features with higher mean importance scores in predicting the y-axis target tend to the left side of the heatmap. Abbreviations: ADLs, activities of daily living; GP general practitioner; IBS, irritable bowel syndrome (-C constipation, -D diarrhoea, -M mixed, -U unclassifiable); LF, lateral flow; PCR, polymerase chain reaction; Sx, symptoms.

## The associations of symptom burden and quality of life

First, a machine model could predict a patient’s health rating with an out-of-sample R² of 0.35 and MAE of 0.34 (Supplementary Figure 1). The top five predictors of personal health rating were, in descending order: i) impact on ADLs, ii) anxiety and depression severity, iii) age, iv) impact on self-care, and v) modified PACQOL. A machine model could predict pain severity with an out-of-sample R² of 0.37 and MAE of 0.33. The five most predictive features of patient-reported pain severity were, in descending order: i) impact on mobility, ii) impact on ADLs, iii) modified PACQOL, iv) personal health rating, and v) anxiety and depression severity. A machine model could also predict anxiety and depression severity with an out-of-sample performance of R^2^ of 0.54 and MAE of 0.38. The five most predictive features of anxiety and depression severity were, in descending order: i) modified PACQOL, ii) impact on ADLs, iii) personal health rating, iv) impact of mental health and wellbeing from the pandemic, and v) impact of bowel symptoms on daily activities. Lastly, a machine model could predict PACQOL with an out-of-sample R² of 0.46 and MAE of 0.37. The five most predictive features of PACQOL were, in descending order: i) impact on ADLs, ii) anxiety and depression severity, iii) impact of bowel symptoms on work, iv) impact of bowel symptoms on daily activity, and v) personal health rating.

## Associations of life impact from functional bowel disorders

A machine model could predict patient employment status with an out-of-sample balanced accuracy of 96% and AUROC 0.96. The five most predictive features of employment status were, in descending order: i) impact of bowel symptoms on daily activities, ii) impact of bowel symptoms on work, iii) impact of mental health and wellbeing from the pandemic, iv) hours of work missed for non-GI reasons, and v) frequency of attendance for bowel symptoms. A machine model could predict the impact of bowel symptoms on daily activities with an out-of-sample R² of 0.67 and MAE of 0.18. The five most predictive features of impact on daily activities from bowel symptoms were, in descending order: i) employment status, ii) modified PACQOL, iii) faecal incontinence, iv) personal health rating, and v) pain severity. A machine learning model can predict the frequency of patient attendance for bowel symptoms with an out-of-sample R² of 0.71 and MAE of 0.28. The five most predictive features of attendance were, in descending order: i) hours of work missed for non-GI reasons, ii) if already seen by a GP, iii) impact of mental health and wellbeing during the pandemic, iv) employment status, and v) if previously seen by a medical consultant. Lastly, a machine learning model could predict the impact of patient mental health on the pandemic with an out-of-sample R² of 0.51 and MAE of 0.26. The five most predictive features of mental health and wellbeing during the pandemic were, in descending order: i) frequency of attendance for bowel symptoms, ii) anxiety and depression severity, iii) impact of sleep from the pandemic, iv) effectiveness of pelvic floor or sphincter exercises, and v) if seen by a GP.

## The associations of treatment effectiveness

Patient-reported effectiveness of laxatives could be predicted with an out-of-sample R² of 0.16 and MAE of 0.57. The five most predictive features of laxative effectiveness were, in descending order: i) effectiveness of footstool use during defaecation, ii) effectiveness of pelvic floor or sphincter exercises, iii) modified PACQOL, iv) effectiveness of change to fluid intake, and v) frequency of attendance for bowel symptoms. The effectiveness of diet changes could be predicted with an out-of-sample performance of R^2^ of 0.37 and MAE of 0.47. The five most predictive features of diet change effectiveness were, in descending order: i) effectiveness of change to fluid intake, ii) pain severity, iii) impact on mobility, iv) frequency of attendance for bowel symptoms and v) effectiveness of footstool use during defaecation. Meanwhile, the effectiveness of footstool use during defecation could be predicted with an out-of-sample R² of 0.18 and MAE of 0.66. The five most predictive features of footstool effectiveness were, in descending order: i) effectiveness of pelvic floor or sphincter exercises, ii) laxative effectiveness, iii) personal health rating, iv) impact of mental health and wellbeing during the pandemic, and v) effectiveness of change to diet.

Patient-reported effectiveness of changes to fluid intake could be predicted with an out-of-sample R² of 0.38 and MAE of 0.45. The five most predictive features of effectiveness in modifying fluid intake were, in descending order: i) effectiveness of change to diet, ii) effectiveness of pelvic floor or sphincter exercises, iii) effectiveness of probiotics, iv) abdominal pain, and v) impact of sleep from the pandemic. The patient-reported effectiveness of pelvic floor or sphincter exercises could be predicted with an out-of-sample R² of 0.41 and MAE of 0.46. The five most predictive features of effectiveness in pelvic floor or sphincter exercises were, in descending order: i) impact of mental health and wellbeing from the pandemic, ii) effectiveness of footstool use during defaecation, iii) effectiveness of fluid intake changes, iv) modified PACQOL, and v) effectiveness of laxatives. Lastly, the patient-reported effectiveness of probiotics, suppositories, and enemas could only be predicted with an out-of-sample performance of R² of 0.10 and MAE of 0.42, R² of 0.08 and MAE of 0.20, and R² of 0.09 and MAE of 0.11, respectively. The five most predictive features of effectiveness in probiotic use were, in descending order: i) effectiveness of change to fluid intake, ii) frequency of attendance for bowel symptoms, iii) effectiveness of change to diet, iv) impact of mental health and wellbeing during the pandemic, and v) pain severity. Relatively few patients reported effects with enemas and suppositories, so we would be cautious about drawing inferences about their predictive features. For completeness, we present these in Figure 6; however, we suggest exercising caution in their interpretation due to the smaller sample size.

## Generative graph community structure

The model entropy (goodness-of-fit criterion) was -635.54 nats after MCMC by simulated annealing, with an iteration curve indicative of model convergence (Supplementary Figure 2).


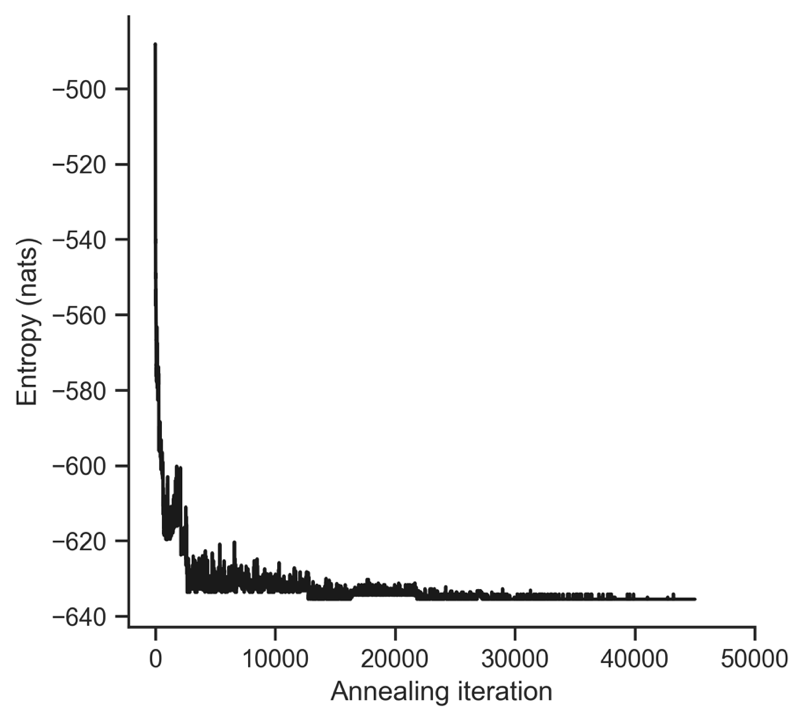


**Supplementary Figure 2.** Line plot of stochastic block model entropy with simulated annealing.

# Methods

**Multivariate imputation through chained equations stability**

We used the standard package for MICE within R, using a single averaged dataset. We increased the default number of imputations and iterations by a factor of ten. This substantially increased the computational time, but was nevertheless undertaken to ensure result stability. The initialisation seed was fixed. To further ensure statistical rigour, we conducted an additional experiment with multiple runs of MICE, varying the random seed. This demonstrated remarkable stability of the dataset, indicative that any downstream impact from imputation parameters would be plausibly negligible (Supplementary Figure 3).


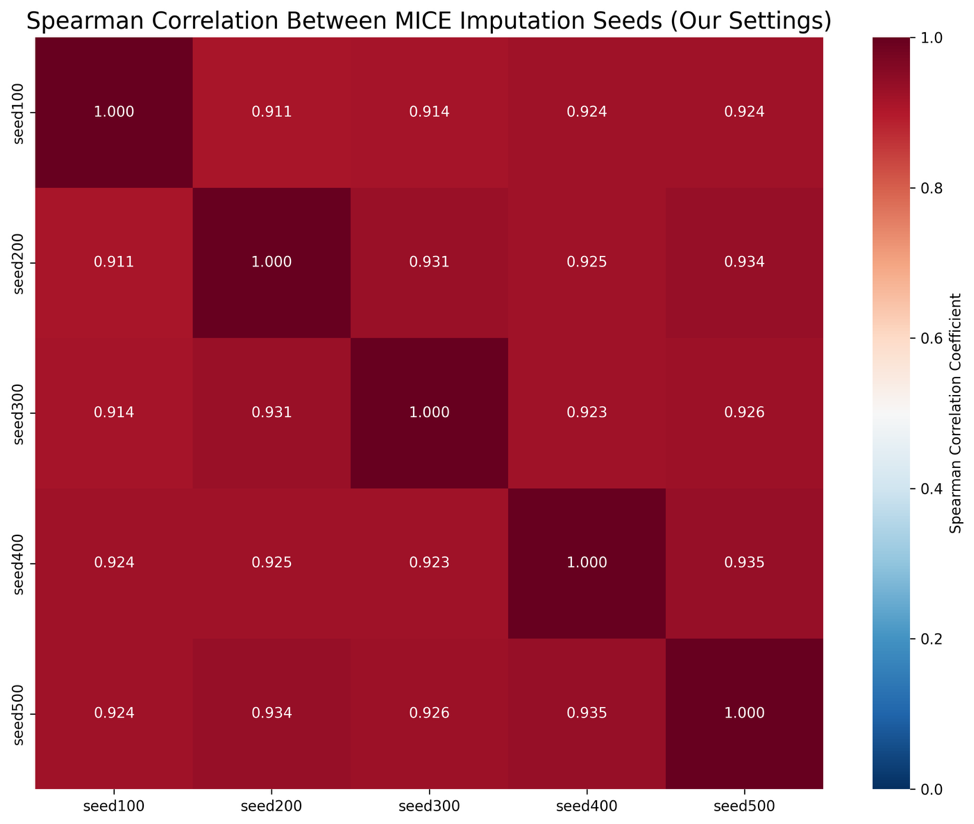


**Supplementary Figure 3**: Imputation stability across different seed randomisations. The heatmap shows dataset comparability regardless of differential seed initialisations.

**Model Initialisation Parameters**

Initial XGBoost models were configured with: Objective: 'reg:squarederror' for regression, 'binary: logistic' for classification; Learning rate: 0.1; Number of estimators: 10; Alpha (L1 regularization): 10; Evaluation metric: 'logloss' for classification; Use label encoder: False; Number of jobs: -1 (all available cores); Tree method: 'gpu_hist'.

**XGBoost Hyperparameter Optimisation**

XGBoost models were optimised using GridSearchCV with 5-fold cross-validation (StratifiedKFold for binary outcomes, standard KFold for continuous outcomes). The following hyperparameter grid was explored: ‘min_child_weight’: [1, 5, 10], ‘gamma’: [0.5, 1, 1.5, 2, 5], ‘subsample’: [0.6, 0.8, 1.0], ‘colsample_bytree’: [0.6, 0.8, 1.0], ‘max_depth’: [3, 4, 5]. This resulted in 135 parameter combinations (3 × 5 × 3 × 3 × 3) evaluated for each model.

**Random Seeds**

The following random seeds were used to ensure reproducibility throughout the remaining analyses:

- Train/test split: random_state=123

- SMOTE oversampling (for binary outcomes): random_state=123

- Multiple imputation (MICE): seed=500

- Cross-validation folds: The results were deterministic, given the fixed random states above**.**
